# Applications of AI to single-cell and spatial transcriptomics: current state-of-the-art and challenges

**DOI:** 10.3389/fbinf.2025.1715821

**Published:** 2026-01-27

**Authors:** Boris Tchatchoua Ngassam, Huilin Niu, Sunny Pang, Valeryia Shydlouskaya, Tallulah S. Andrews

**Affiliations:** 1 Department of Biochemistry, Schulich School of Medicine and Dentistry, University of Western Ontario, London, ON, Canada; 2 Department of Microbiology and Immunology, Schulich School of Medicine and Dentistry, University of Western Ontario, London, ON, Canada; 3 Department of Computer Science, University of Western Ontario, London, ON, Canada

**Keywords:** cell-cell interactions, cross-dataset integration, data denoising, deconvolution, dimensionality reduction, integrating single-cell and spatial transcriptomics modalities, transcriptional velocity

## Abstract

Artificial intelligence (AI) has become a common tool for bioinformatics, with hundreds of methods published in recent years. Due to the training data demands of deep-learning algorithms, high-throughput single-cell and spatial transcriptomics is one of the most popular areas for these applications. Here we review how AI is being used for single-cell and spatial transcriptomics analysis, and how these approaches compare to alternative statistical or heuristic-based methods. We explored 10 common analysis tasks: dimensionality reduction, cross-dataset integration, data denoising, data augmentation, deconvolution, cell-cell interactions, transcriptional velocity, transcriptomic-chromatin accessibility integration, and integrating single-cell and spatial transcriptomics modalities. We highlight which algorithms are likely to be useful for discovery researchers, and which are not yet ready for general research use.

## 1 Introduction

Artificial intelligence (AI) has revolutionized the analysis of big data across many fields, including biomedical research, and is entering clinical practice, with over 1,000 algorithms and devices approved by the FDA (Health, 2025). While the predominant use of AI in clinical practice is in biomedical image analysis, in research, AI approaches have gained increasing popularity in bioinformatics, and especially single-cell and spatial transcriptomics (Ge et al., 2024; Erfanian et al., 2023; Zahedi et al., 2024; Molho et al., 2024; Ma and Xu, 2022). AI is often used synonymously or as a subtopic of the broader field of machine learning. Machine learning involves a computer or algorithm deriving at least some aspects of a model from observed or “training” data. This includes tasks as simple as estimating the slope and intercept of the best-fit line, or those as complex as labelling MRI images with specific pathological lesions. AI, or deep learning (DL) as we will refer to it, is a specific class of models based on neural networks (NN) with multiple interconnected layers of functions capable of learning complex, non-linear patterns within large-scale datasets.

**FIGURE 1:**
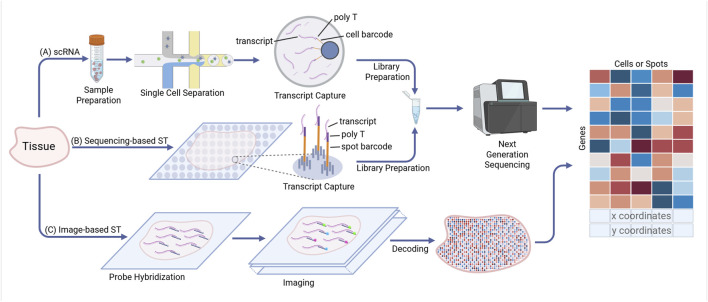
Single cell and spatial transcriptomics workflow. **(A)** Droplet-based single cell RNA sequencing. Tissue is dissociated into single cells which are co-encapsulated with barcoded beads by microfluidics. Released transcripts are captured by poly T and sequenced following library preparation. **(B)** Sequencing-based ST. A tissue section is placed on a slide with spatially barcoded capture spots. Transcripts are captured and sequenced following library preparation. **(C)** Image-based ST. Transcripts are hybridized with fluorescence probes and imaged over multiple rounds. After images decoding and cell segregation, each fluorescent dot represents an individual transcript. All methods produce gene expression, with spatial methods providing additional x, y coordinates for downstream analysis.

Single-cell and spatial transcriptomics are especially amenable to DL due to the large number of observations, as most datasets consist of thousands to millions of individual cells and thousands to tens of thousands of transcripts (Svensson et al., 2018). State-of-the-art single-cell transcriptomics (scRNA-seq) experiments typically generate large-scale datasets composed of 20,000–500,000 individual cells from at least three samples from one or more conditions (Figure 1A). These data undergo quality control, normalization, dimensionality reduction, integration across samples or across modalities, then they are clustered and annotated with cell type labels based on the expression of characteristic genes (Heumos et al., 2023; Luecken and Theis, 2019; Andrews and Hemberg, 2018; Kiselev et al., 2019). Many of these tasks are classic machine learning problems which could potentially be performed by DL models. Spatial transcriptomics (ST) adds two additional layers of information: two-dimensional coordinates of each cell, which may soon to be three-dimensional (Schott et al., 2024), as well as one or more layers of histology (H&E) and/or immunofluorescent (IF) images of the tissue. ST comes in two main types: sequencing-based (Figure 1B) and imaging-based (Figure 1C). In imaging-based ST, transcripts are individually measured with single-molecule fluorescent *in situ* hybridization (Chen et al., 2015; He et al., 2022) (Figures 1B,C). Transcripts are aggregated at the level of individual cells by identifying nuclei and cell boundaries, referred to as tissue-segmentation or simply segmentation (Mitchel et al., 2025; Polański et al., 2024). In many cases, this single-cell resolution ST data is analyzed using the same tools developed for scRNAseq. For sequencing-based ST, tissue is placed on a slide covered in oligonucleotide spots which capture and tag transcripts with a spatial barcode. Resolution is determined by the size of each uniquely barcoded spot. In many cases, these spots will overlap more than 1 cell, thus requiring “deconvolution” to estimate the contribution of each cell to the transcripts captured by that spot (Ståhl et al., 2016; Rodriques et al., 2019; Gaspard-Boulinc et al., 2025). For both approaches, but particularly for sequencing-based techniques, information from the matching images can be combined with transcriptomics to improve the identification of distinct anatomical regions either in parallel with or integrated into the ST analysis workflow (Williams et al., 2022; Pham et al., 2023; Zhao et al., 2021). Tissue segmentation and extraction of biologically relevant features from tissue imaging is dominated by DL algorithms (Chen et al., 2024; Stringer et al., 2021; Warren and Moustafa, 2023; Kuntz et al., 2021; Greenwald et al., 2022).

While these technologies have generated large amounts of high-dimensional datasets, the analysis of these data is challenged by a combination of biological complexity and technical noise. Biologically, cellular states exist along continuous trajectories—such as differentiation or activation—and exhibit high heterogeneity within and across tissues. Technically, the data is affected by low sensitivity, batch effects, ambient RNA contamination, and spatial blur in low-resolution spatial assays (Ge et al., 2024; Kiselev et al., 2019; Mitchel et al., 2025; Lähnemann et al., 2020; Young and Behjati, 2020; Svensson et al., 2017). These factors introduce spurious variation, obscure true biological signals, and complicate tasks such as clustering, integration, and cell–cell communication inference.

In recent years, DL has emerged as a novel approach to address the computational challenges of scRNA-seq and ST. These methods excel at feature extraction and classification of high-dimensional, noisy data, thus making them well-suited for cell type annotation, multimodal data integration, and nonlinear dimensionality reduction (Erfanian et al., 2023; Karin et al., 2024; Sarker, 2021). DL methods can take advantage of GPU, parallel computing, and iterative optimization on batches of data to scale analyses to datasets of millions of observations; however, similar or better performance can also be achieved by optimizing classical statistical methods (Chockalingam et al., 2025). DL models are extremely flexible and can be combined to allow for the joint analysis of multiple data types such as integration of scRNA-seq and ST data, or imaging and transcriptomic data.

In recent years, there has been an explosion of methods developed for scRNA-seq and ST analysis using DL models (Table 1). Despite their growing number, only a few have achieved broad adoption in the research community. While existing reviews (Zahedi et al., 2024; Ma and Xu, 2022; Li Y. et al., 2022; Era et al., 2019; Luo et al., 2024; Wani et al., 2025) have primarily focused on the technical aspects of these models, their architecture, and training strategies, we focus instead on their performance in biological discovery research and on which, if any, of these tools have been shown to enhance accuracy, reproducibility, and sensitivity for biological discovery. As such, we first provide a brief overview of different model architectures, then discuss DL approaches to addressing specific bioinformatics analysis tasks, and their applicability to real-world discovery research. This will help biologically focused researchers understand when and how to use these methods and help bioinformaticians determine which tasks are appropriate for DL models and how to evaluate their design to ensure the resulting model is useful to the biomedical research community.

**TABLE 1:** Deep learning-based tools in single-cell RNA sequencing and spatial transcriptomics.

Tool name	Task category	Modality	DL model	Key features/notes	Year	Code
SCANVI	Annotation/transfer	scRNA-seq, ST	Conditional VAE	Semi-supervised cell labeling. Exetends scVI for annotation	2021	Code
BBKNN	Batch correction	scRNA-seq	Graph-KNN	Batch integration	2020	Code
BERMUDA	Batch correction	scRNA-seq	AE + clustering	Deep AE-based alignment	2019	Code
scArches	Batch correction/integration	scRNA-seq	VAE w/fine-tuning	Architectural surgery for model reuse	2021	Code
trVAE	Batch correction/integration	scRNA-seq	Conditional VAE	Domain transfer using adversarial training	2020	Code
scGEN	Batch correction/integration	scRNA-seq	VAE	Predicts perturbed gene states	2019	Code
Graphcomm	Cell-cell interaction	scRNA-seq	GAT	Integrates multimodal data for cell-cell communication	2025	Code
scSDNE	Cell-cell interaction	scRNA-seq	GNN + AE	Semi-supervised graph embedding integrating ligand-receptor and gene regulation data	2025	Code
DeepCCI	Cell-cell interaction	scRNA-seq	GCN + ResNet	Supervised cell–cell interaction network prediction using L–R pairs	2023	Code
scTenifoldXct	Cell-cell interaction	scRNA-seq	Neural networ + semi-supervised, manifold alignment	Predicts cell-cell interactions and maps communication graphs using ligand-receptor gene embedding and manifold alignment	2023	Code
Spatialscope	Cell-cell interaction	Spatial transcriptomics	Deep generative model	Decomposes ST spots to single cells using generative models	2023	Code
CellFM	Cell type annotation	scRNA-seq	RetNet	Foundamental model for annotation	2025	Code
scAtlasVAE	Cell type annotation	scRNA-seq	VAE	Cross atlas comparison and transfer learning for cell subtype annotation	2024	Code
scGAA	Cell type annotation	scRNA-seq	Transformer	Combines horizontal and vertical attention mechanisms, does not require batch information	2024	Code
TOSICA	Cell type annotation	scRNA-seq	Transformer	Combine cell type marker genes and transformer attention layers	2023	Code
scBERT	Cell type annotation	scRNA-seq	Transformer	Pretrained on gene expression	2022	Code
SIMS	Classification	scRNA-seq	Transformer	Uses TabNet transformer for lable transfer from cell atlas	2024	Code
expiMap	Classification	scRNA-seq	AE w/pathway constraints	Maps cells to known pathways in a reference dataset	2023	Code
scDLC	Classification	scRNA-seq	LSTM + DNN	Sequential modeling for classification	2022	Code
SEDR	Clustering	Spatial transcriptomics	VGAE	Integrates latent of GE + spatial embedding	2024	Code
SiGra	Clustering	Spatial transcriptomics	Graph transformer	Integrates multichannel images + expression	2023	Code
SpaGCN	Clustering	Spatial transcriptomics	GCN	Uses spatial coordinates + histology + GE	2021	Code
scDCC	Clustering	scRNA-seq	AE	Semi-supervised with pairwise constraints	2021	Code
scVAE	Clustering	scRNA-seq	VAE	Use VAE to learn low dimensional representation to facilitate accurate clustering	2020	Code
scDeepCluster	Clustering	scRNA-seq	AE	Unsupervised clustering with deep autoencoder	2019	Code
GSI	Clustering	Spatial transcriptomics	VAE	Integrates image + GE + spatial coordinates to improve clustering	2025	Code
Deep scSTAR	Clustering/Annotation/Embedding	scRNA-seq	DAE + MLP + MTL	Denoising autoencoder with supervised MLP in latent space	2025	Code
SAUCIE	Clustering/Batch corrcetion	scRNA-seq	AE	Use maximal mean discrepancy penalty to match distributions of batches	2019	Code
STAGATE	Clustering/batch correction	Spatial transcriptomics	GAT	Adaptive graph attention on spots	2022	Code
SPADE	Clustering/deconvolution	Spatial transcriptomics	spaGCN + Lasso regression	H&E img + GE for clustering, then uses ref scRNAseq data for domain deconvolution	2024	Code
SPACEL	Clustering/deconvolution	Spatial transcriptomics	VAE + GCN	Self-supervised local clustering + simulation	2023	Code
SpaCell	Clustering/embedding	ST + histology	AE + CNN	AE model for embeddings and CNN for classification	2020	Code
scResolve	Deconvolution	Spatial transcriptomics	Transformer + VAE	Reference-free, integrate cell segmentation of histology image	2024	Code
UniCell Deconvolve	Deconvolution	Spatial transcriptomics + bulk RNA seq	Deep feedforward network	Foundamental model	2023	Code
DAISM-DNNXMBD	Deconvolution	Bulk RNA seq	DNN	Train 1 DNN for each cell type	2022	Code
Tangram	Deconvolution	Spatial transcriptomics	Custom model	Custom probablistic model + gradient descent optimization + backpropagation	2021	Code
DSTG	Deconvolution	Spatial transcriptomics	CCA + MNN + GCN	Graph reconstruction	2021	Code
Scaden	Deconvolution	Bulk RNA seq	DNN	Ensemble of three best-permorning DNN	2020	Code
scAR	Denoising	scRNA-seq	VAE	Ambient RNA denoising	2022	Code
DCA	Denoising/imputation	scRNA-seq	Autoencoder	Deep count autoencoder (NB/ZINB)	2018	Code
STGNNks	Embedding	Spatial transcriptomics	GAE	Graph-based clustering	2023	Code
scSemiProfiler	Embedding	scRNA-seq	VAE-GAN + active learning	Learns cell states *via* active bulk supervision	2023	Code
scGNN	Embedding/clustering	scRNA-seq	GNN	Graph-based denoising, clustering, embedding	2021	Code
scVI	Embedding/imputation/integration	scRNA-seq, ST	VAE	Probabilistic latent space, batch correction	2018	Code
scGFT	Generation	scRNA-seq	GAN, VAE, GFT	Generate synthetic scRNA seq data that reflects natural biological variability	2025	Code
STAGE	Generation	Spatial transcriptomics	AE	Data generation	2024	Code
scCross	Generation	scRNA-seq	VAE + GAN + MNN	Cross-domain latent space used for simulation	2024	Code
SRTsim	Generation	Spatial transcriptomics	Empirical sim	Simulates spot-based ST data	2023	Code
cscGAN	Generation	scRNA-seq	Conditional GAN	Cell type aware generator	2020	Code
scIGANs	Imputation	scRNA-seq	GAN	Conditional GAN	2020	Code
DeepImpute	Imputation	scRNA-seq	DNN	Imputation using sub-neural network modules	2019	Code
autoCell	Imputation/feature extraction	scRNA-seq	Graph-enhanced VAE	Uses VAE and GNN	2023	Code
scGPT	Integration	scRNA-seq	Transformer	Foundational model	2024	Code
SpatialGLUE	Integration	ST + proteinmics + epigenomics	AE + graph fusion	Integrate the different omics modalities with spatial information	2024	Code
MultiVI	Integration	scRNA + ATAC	VAE	Joint ATAC–RNA modeling	2023	Code
SCALEX	Integration	scRNAseq	Encoder + GAN	Use feature links to preserve biological variation	2021	Code
spaVAE	Low-dimensional space	Spatial transcriptomics	VAE	NB model based VAE, combining Gaussian process prior and Gaussian prior	2024	Code
COVET	Low-dimensional space	scRNA-seq, ST	ENVI + CVAE	Encode the covariance of gene expression between neighboring cells joint latent space	2024	Code
scMODAL	Multimodal integration	scRNA-seq + ATAC	Multimodal AE + GAN	Use feature links to align cell embeddings	2025	Code
scMVP	Multimodal integration	scRNA-seq + scATAC-seq	Multi-view VAE	Handles paired multi-omics, encodes ATAC with attention, integrates views for embedding and clustering.	2022	Code
GLUE	Multimodal integration	scRNA-seq, scATAC-seq, snmC-seq	VAE per modality + graph linking features	Models regulatory feature interaction across modalities; scalable large-dataset integration.	2022	Code
totalVI	Multimodal integration	scRNA-seq + protein	VAE	Probabilistic multimodal model	2021	Code
Cobolt	Multimodal integration	scRNA-seq + scATAC-seq (and others)	Multimodal VAE	Integrates joint and single-modality datasets.	2021	Code
scButterfly	Multimodal integration	scRNAseq + ATAC	U-net + AE	Image-guided gene embedding	2024	Code
Monae	Multimodal integration	scRNAseq + ATAC	AE + Contranstive learning	Modality-specific auto-encoders	2024	Code
MIDAS	Multimodal integration/Batch correction/Embedding	scRNAseq + ATAC + ADT (proteomics)	AE	Self supervised modality alignment, transfer learning	2024	Code
BIDCell	Self-supervised learning	Spatial transcriptomics	AE + biologically-informed loss	Learns spatial gene-region relationships	2023	Code
STAGNN	Spatial clustering	Spatial transcriptomics	GAT	Graph attention network (GAT) and the time series model informer	2024	Code
TransformerST	Spatial domain clustering	Spatial transcriptomics	ViT + adaptive graph transformer	Uses H7E image features and GE in self-attention transformer	2024	Code
DeepST	Spatial domain detection	Spatial transcriptomics	Multi-stage deep learning using DNN, VGAE	Image + gene-based spatial clustering	2022	Code
GIST	ST integration	Spatial transcriptomics	CNN + graph transformer	Uses GE + cell type-informative paired tissue images e.g., IF	2022	Code
DeepVelo	Trajectory/RNA velocity embedding	scRNA-seq	GCN + DNN	Models gene- and cell-specific transcriptional kinetics	2024	Code
VeloVI	Trajectory/RNA velocity embedding	scRNA-seq	VAE	Learns gene-specific kinetics, provides uncertainty quantification for velocities; flexible for time-dependent transcription rates.	2023	Code
cellDancer	Trajectory/RNA velocity embedding	scRNA-seq	DNN	Predicts cell- and gene-specific transcription, splicing and degradation rates	2023	Code
VeloVAE	Trajectory/RNA velocity embedding	scRNA-seq	VAE	Extends velocity modelling with VAE framework to capture kinetic variability.	2022	Code
LatentVelo	Trajectory/RNA velocity embedding	scRNA-seq	VAE/latent emberdding model	Learns latent representation for velocity; enables batch correction and dynamics embedding.	2022	Code
VeloAE	Trajectory/RNA velocity embedding	scRNA-seq	AE	Embeds velocity information (spliced/unspliced) for better dynamic modelling.	2021	Code

## 2 Common deep learning architecture

### 2.1 Convolutional neural networks (CNN)

**FIGURE 2:**
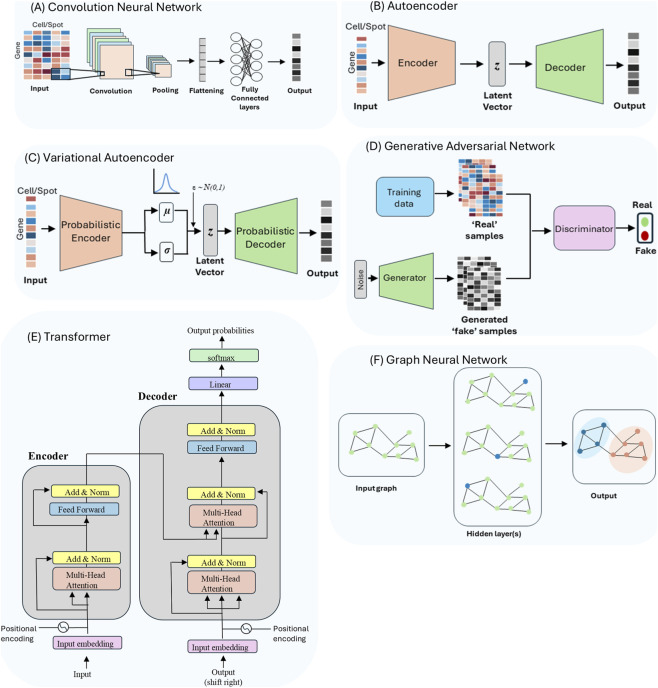
Deep learning architectures commonly applied to single-cell and spatial transcriptomics. **(A)** Convolutional neural network (CNN): extracts local spatial patterns from image-like inputs (e.g., cell/spot × gene maps) *via* convolution–pooling stacks. **(B)** Autoencoder (AE): learns a low-dimensional latent vector (z) that reconstructs the input, enabling denoising and feature learning. **(C)** Variational autoencoder (VAE): probabilistic AE that learns a distribution over (z) (parameterized by (µ, σ) and samples (z + ε ∼ N (0,1)) for generative modeling. **(D)** Generative adversarial network (GAN): a generator synthesizes expression profiles from noise while a discriminator distinguishes real from generated samples. **(E)** Transformer tokenizes inputs and applies positional embeddings with stacked self-attention and feed-forward blocks in an encoder to produce task-specific outputs. **(F)** Graph neural network (GNN): propagates information over a cell/spot graph to model neighborhood structure and produce node-level outputs.

Convolutional neural networks (CNN) were originally developed for structured data in the form of multiple arrays, such as images which are composed of pixel intensities in 2D arrays for each color channel (Lecun and Bengio, 1998). Their design is built around three core principles (Lecun and Bengio, 1998): (i) local receptive fields, which focus computation on neighboring input values to capture features such as edges and corners in images; (ii) shared weights, which enable the same filter to be applied across inputs, thereby reducing the number of parameters; and (iii) subsampling or pooling operations, which introduce robustness of outputs to distortions and shifts. Together, these principles allow CNNs to efficiently recognize local patterns and build hierarchical feature representations using fewer parameters than fully connected networks (Figure 2A). Due to these advantages, CNNs have become a popular architecture in fields such as computer vision, where extracting informative features from local patterns is crucial.

Although scRNA-seq lacks inherent spatial structure, gene expression data has been successfully adapted by restructuring it into an image-like format used by CNNs. A method called convolutional neural network for co-expression (CNNC) encodes gene pair co-expression as 2D histograms, which serve as input “images” (Yuan and Bar-Joseph, 2019). This approach allows CNNs to learn complex, nonlinear gene-to-gene relationships directly from single-cell expression data. CNNs are particularly valuable for ST to extract morphological features from tissue sections that complement transcriptomics data. Methods such as SpaCell (Tan et al., 2020) combine pretrained CNN models with an autoencoder network to learn joint embeddings of histology and gene expression. Similarly, stLearn (Pham et al., 2023) leverages a pretrained CNN model to extract morphological features from histology images and integrates them with gene expression data to map spatial domains within tissue sections.

### 2.2 Autoencoders (AE)

Autoencoders (AE) are deep feed-forward neural networks fundamentally designed for unsupervised representation learning, where the goal is to learn lower-dimensional features of high-dimensional data. Structurally, an AE consists of an encoder network and a decoder network (Figure 2B). The encoder compresses input data (such as gene expression vector from a cell) into a lower-dimensional latent space, while retaining the most significant features. The decoder, which typically mirrors the architecture of the encoder, aims to reconstruct the high-dimensional input data from the learned low-dimensional representation. The entire network is trained to minimize the reconstruction error given as the mean squared error between input and reconstructed data. The resulting latent representations, also called embeddings, are particularly valuable as they serve as nonlinear counterparts to traditional linear dimensionality reduction techniques such as Principal Component Analysis (PCA). While popular pipelines like Seurat (Stuart et al., 2019; Satija et al., 2015; Butler et al., 2018) use PCA and assume linear relationships among genes, AEs can capture complex nonlinear relationships inherent in scRNA-seq data. A key advantage of AEs lies in their flexibility to adapt the reconstruction objective based on the statistical properties of the data. For instance, loss functions can use negative binomial or zero-inflated negative binomial distributions, which are appropriate for single-cell and spatial transcriptomics data (BinTayyash et al., 2021; Svensson, 2020; Zhao et al., 2022) instead of standard statistics such as mean squared error (MSE), which assume Gaussian noise. This way, AE can incorporate probabilistic assumptions directly into the loss function by modeling the likelihood of an appropriate probability distribution. The model can then account for data-specific characteristics such as sparsity, overdispersion, and technical noise commonly observed in scRNA-seq data, hence learning more biologically meaningful representations that respect the underlying statistical structure of gene expression measurements.

In scRNA-seq analysis, Deep Count Autoencoders (DCA) leverage the flexibility of AE by modeling the output as the parameters of the zero-inflated negative binomial distribution (Eraslan et al., 2019), commonly used for RNA-seq counts (Svensson, 2020). Additionally, prior domain knowledge can be incorporated into an AE in a semi-supervised training manner as implemented by scDCC (Single Cell Deep Constrained Clustering) (Tian et al., 2021). scDCC integrates soft pairwise constraints derived from prior biological information (marker genes or cell type annotation) into the model’s loss function. These constraints guide the model to group related cells and separate dissimilar ones during latent space optimization, effectively shaping the embedding to reflect domain knowledge. This approach improves clustering accuracy and biological relevance, especially in complex or noisy datasets, showcasing autoencoders as versatile frameworks for single-cell data analysis.

### 2.3 Variational autoencoders

Variational autoencoders (VAEs) are a probabilistic extension of standard AEs, designed to improve representation learning and generative modeling by incorporating principles of Bayesian inference to learn a distribution over a latent (lower-dimensional) space. This probabilistic formulation addresses a key limitation of AEs: their deterministic latent space, which often results in discontinuous or overfitted representations that generalize poorly to unseen data and lack support for structured sampling (Kingma and Welling, 2022; Doersch, 2021; Kingma and Welling, 2019; Rezende et al., 2014). Despite their architectural similarity, VAEs differ fundamentally in that they encode each input to the parameters of a probability distribution (usually Gaussian) from which a latent variable is sampled (Figure 2C). The decoder reconstructs the input data from this latent representation. This formulation enables VAEs to learn smooth, continuous, and structured latent representations by optimizing a joint loss function composed of a reconstruction term and a Kullback-Leibler (KL) divergence term, which regularizes the approximate posterior distribution to be close to the prior distribution. The key advantage of VAEs lies in their ability to model data uncertainty and support generative capabilities through a probabilistic latent space. This is particularly valuable for scRNA-seq, where modeling sparsity, overdispersion and technical noise is essential (Svensson, 2020).

Models such as scVI (Lopez et al., 2018) (Single-Cell Variational Inference) build upon the VAE framework to model scRNA-seq count data using a negative binomial likelihood, while simultaneously correcting for batch effects. Similarly, totalVI (Gayoso et al., 2021) extends the VAE architecture to jointly model RNA and protein data from CITE-seq (cellular indexing of transcriptomes and epitopes by sequencing), enabling multimodal inference (Stoeckius et al., 2017). Concretely, totalVI places a logistic-normal prior on a shared cell-level latent representation that parameterizes modality-specific likelihoods by using a negative binomial RNA counts and a negative-binomial mixture for proteins, respectively. In ST, SpaVAE (Tian et al., 2024) incorporates spatial coordinates *via* a Gaussian process prior on the latent space that is indexed by the spot coordinates while keeping some latent dimensions under the standard gaussian prior to capture non-spatial spot variations. In general, VAEs are flexible in that different likelihoods can be used and latent priors can also be customized to encode known structure in the data such as spatial information and batch effects.

### 2.4 Generative adversarial networks (GANs)

Instead of learning to reconstruct what already exists, GANs learn by deception (Goodfellow et al., 2014). They consist of a generator, which creates synthetic data from random noise, and a discriminator, which attempts to distinguish between real and generated samples (Figure 2D). Through adversarial training, the generator improves its ability to produce realistic outputs, while the discriminator becomes more adept at detecting “fake” or synthetic data. This dynamic results in a generator that can synthesize high-quality, biologically plausible gene expression profiles.

In scRNA-seq, cscGAN/scGAN (Marouf et al., 2020) learns to generate cell type conditioned expression profiles that preserve gene–gene dependencies, supporting augmentation of rare populations and improving downstream classification and clustering. scIGAN (Xu et al., 2020) frames imputation as generation, using an adversarial loss (often combined with count-aware objectives) to recover missing values while retaining biological variability in different cell types. Adversarial alignment has also been used for batch/platform correction. For instance, iMAP (Wang D. et al., 2021) couples an autoencoder backbone with a GAN discriminator that removes batch signal from the latent space, enabling cross-platform integration of tumor microenvironment datasets while preserving cell-state structure.

GANs are widely used in digital pathology for histology image generation and translation, demonstrating strong capability on imaging. However, in ST there is still no widely adopted, end-to-end GAN framework that jointly models histology images, gene expression, and spatial coordinates. Challenges such as training instability, mode collapse, and lack of biological interpretability make it difficult to ensure that generated spatial gene expression patterns reflect true biological variation rather than technical artifacts. As a result, GANs are not standard components of ST analysis pipelines, where AE, VAEs, GNNs, and transformers currently dominate.

### 2.5 Transformer

Transformers are deep learning models originally developed for natural language processing (NLP) with an encoder-decoder architecture composed of self-attention layers (Vaswani et al., 2023) (Figure 2E). Although they are similar to AEs in design, they differ in several aspects. The encoder and decoder can be trained and used individually, as seen in models used by BERT and GPT respectively (Yenduri et al., 2023; Devlin et al., 2019). The self-attention layers dynamically integrate each input element with all elements within the same input sequence, capturing contextual relationships. Additionally, the encoder is not constrained by a low-dimensional latent space, and the decoder is usually trained to autoregressively generate a target sequence rather than reconstruct the input (Vaswani et al., 2023; Xiong et al., 2025). These properties have made transformers the backbone of modern foundational models, which are pretrained on large and heterogeneous datasets and then adapted to a wide range of downstream tasks with minimal supervision.

Transformers have driven significant advances in modeling sequential data in domains like natural language processing (Wu et al., 2025), time-series analysis (Wen et al., 2023), and DNA (Avsec et al., 2021) and protein sequences (Rives et al., 2021), for which they were originally designed. Transcriptomics data is inherently non-sequential and requires the encoding of gene expression values into token-like embeddings, analogous to tokens in NLP, which transformers can process. Current approaches vary in how they represent expression levels, each with distinct advantages and limitations. One approach is ordering, where genes are ranked by transcript abundance within a cell and treated as an ordered sequence of tokens, with each gene assigned a learned embedding (Levine et al., 2024), as implemented by tGPT (Shen et al., 2023), iSEEK (Shen et al., 2022), GeneMamba (Qi et al., 2025), and Geneformer (Theodoris et al., 2023). While this method captures relative patterns and is more robust to technical noise and batch effects (Shen et al., 2023; Qi et al., 2025), quantitative expression information is lost during data transformation (Levine et al., 2024), resulting in reduced data resolution. A second approach is bin-based discretization, where gene counts are grouped into predefined bin sizes, each with an assigned learnable embedding (Yang et al., 2022; Cui et al., 2024). Although the absolute scale of expression is preserved and sequence modeling is simplified, fine-grained biological signal is lost, particularly for genes with subtle but functionally relevant expression differences, which can be sensitive to bin boundaries and potentially affect downstream analysis. Alternatively, the value projection strategy avoids discretization altogether by directly mapping gene expression values to a learnable embedding, which is combined with a gene-specific embedding (Hao et al., 2024a; Zeng et al., 2025), resulting in a transformer input token. This retains the full resolution of the original data and avoids artifacts due to discretization.

In ST, transformers’ ability to take multimodal input and model long range dependencies offers distinct advantages over other methods (Xu P. et al., 2023; Hao et al., 2024b; Wen et al., 2024). In contrast to local neighborhood-based approaches such as GNN or clustering algorithms, that focus on immediate spatial proximity, transformers can capture global spatial relationships across tissue sections through self-attention.

### 2.6 Graph neural networks

Graph Neural Networks (GNNs) are deep learning models designed to operate on graph-structured data, where entities are represented as nodes and their relationships as edges (Figure 2F). Unlike architectures that treat samples as independent vectors, GNNs iteratively update node representations by aggregating information from their neighbors, making them well suited to capture community structure, dependencies, and spatial organization. This is particularly relevant for single-cell and spatial transcriptomics, where cells can be connected by transcriptional similarity, gene co-expression networks, or spatial spots by physical adjacency.

A key strength of GNNs is that they operate directly on graphs while integrating with other deep models, which improves representation learning for biological data. Graph Convolutional Networks (GCNs) extend convolution to cell–cell graphs and enable semi-supervised label transfer. scGCN (Song et al., 2021) builds a hybrid graph that links reference and query datasets through mutual-nearest-neighbor connections in a shared low-dimensional space and augments it with within-query neighbors. A GCN then propagates labels across this graph using variable-gene features, aligning matched cells and flagging unlabeled cells. In ST, SpaGCN (Hu et al., 2021) constructs a weighted spatial graph that combines spot proximity, histology image features and gene expression similarity and then uses a GCN to learn spot representations for tissue domain detection.

Beyond CNNs, GNNs have been incorporated into standard and variational AE frameworks to enable representation learning guided by transcriptomic similarity and spatial proximity. Models such as GVAE (Graph Variational Autoencoders) (Simonovsky and Komodakis, 2018) integrate GNNs with VAEs, leveraging the generative capacity of VAEs together with graph-based regularization. In scRNA-seq, graph-sc (Ciortan and Defrance, 2022) uses a graph autoencoder framework to learn low-dimensional embeddings used for clustering, while scGNN (Wang J. et al., 2021) extends this approach by reconstructing both gene expression and cell similarity graph structures. More recently, self-attention has been incorporated into GNN, giving rise to Graph Attention Networks (GATs) that learn edge-specific weights during neighborhood aggregation instead of averaging contributions equally from all neighbors as in GCNs (Veličković et al., 2018). STAGATE (Dong and Zhang, 2022) adapts this approach with a graph-attention autoencoder on the spatial neighbor network, where self-attention layers learns edge-specific weights normalized with softmax which are then used to update spot specific representations. In contrast, GraphST (Long et al., 2023) employs a GNN encoder with contrastive learning on the spatial graph, encouraging nearby neighbors map to similar representations and forcing distant spots to map to dissimilar ones. This contrastive formulation yields representations that are more robust to noise and batch effects, thereby improving domain separation as well as downstream clustering.

### 2.7 Hybrid models

Recent advances in deep learning for single-cell and spatial transcriptomics have led to the development of hybrid models that combine the strengths of multiple architectures to address complex, multimodal challenges. These models integrate components from different frameworks such as VAEs, GANs, GNNs, and Transformers to capture diverse aspects of biological data, including nonlinear dependencies, spatial structure, temporal dynamics, and multimodal relationships. Unlike monolithic architecture, hybrid models are designed to be modular and flexible, enabling tailored solutions for specific biological questions.

One common hybrid design combines VAEs and GANs, leveraging the probabilistic latent space of the VAE for structured representation learning and the adversarial refinement of the GAN for improved sample generation. iMAP (Wang D. et al., 2021) (AE + GAN) exemplifies this approach by using a GAN to align latent spaces across batches.

Another combination integrates GNNs with VAEs (different from GVAE), where the GNN captures spatial or transcriptional neighborhood information, and the VAE provides a probabilistic and generative framework. For instance, scGNN (Wang J. et al., 2021) combines graph-based message passing with autoencoding to jointly reconstruct gene expression and preserve cell-cell similarity.

More recently, hybrid models have incorporated transformers and GNNs, merging global attention with local graph structure. STAGATE (Dong and Zhang, 2022) uses a GAT to model spatial dependencies, effectively combining the neighborhood aggregation of GNNs with the weighted feature integration of attention. This allows the model to identify both local tissue domains and long-range functional relationships. These hybrid approaches demonstrate that the future of deep learning in genomics lies not in isolated architecture, but in strategic integration, where each component addresses a specific biological or technical challenge. By combining the generative power of VAEs, the spatial awareness of GNNs, the global context of transformers, and the realism of GANs, hybrid models offer a more comprehensive and interpretable framework for analyzing the complexity of single-cell and spatial data.

## 3 Applications of DL to scRNA-seq and ST analysis tasks

Most methods utilize unsupervised models, which do not require any “ground truth” or predetermined labels for the training data. This enables these methods to be trained on each individual experiment, customizing the model for each application. Alternatively, DL models can be pretrained on hundreds to thousands of datasets of a similar type to create a generalizable ‘foundation’ model (Chen et al., 2024; Heimberg et al., 2025). For example, the UNI foundation model of pathology images was trained on over 100,000 individual images (Chen et al., 2024), whereas stLearn (Pham et al., 2023) and scVI (Lopez et al., 2018) retrain their NNs to extract dataset-specific features. In contrast, supervised models require training data with a known ground truth answer for the specific task it is designed to perform. Most often, these models involve classification, such as stDeepSort, which was trained on various reference datasets to annotate cell types in single-cell data (Shao et al., 2021), or Cellpose, trained to recognize and segment cells based on thousands of manually labelled training images (Stringer et al., 2021).

The most common use of DL when analyzing high dimensional data, such as scRNA-seq and ST, is to learn a lower dimensional embedding space, conceptually similar to principal component (PCA) space but without the assumptions and constraints. This embedding space can then be used for a variety of tasks either within the DL framework or extracted and used in standard statistical analysis as a replacement for PCA. Here we will discuss the main approaches to generating DL embeddings and their application for scRNA-seq and ST data.

### 3.1 Dimensionality reduction, clustering, and spatial domain identification

**FIGURE 3:**
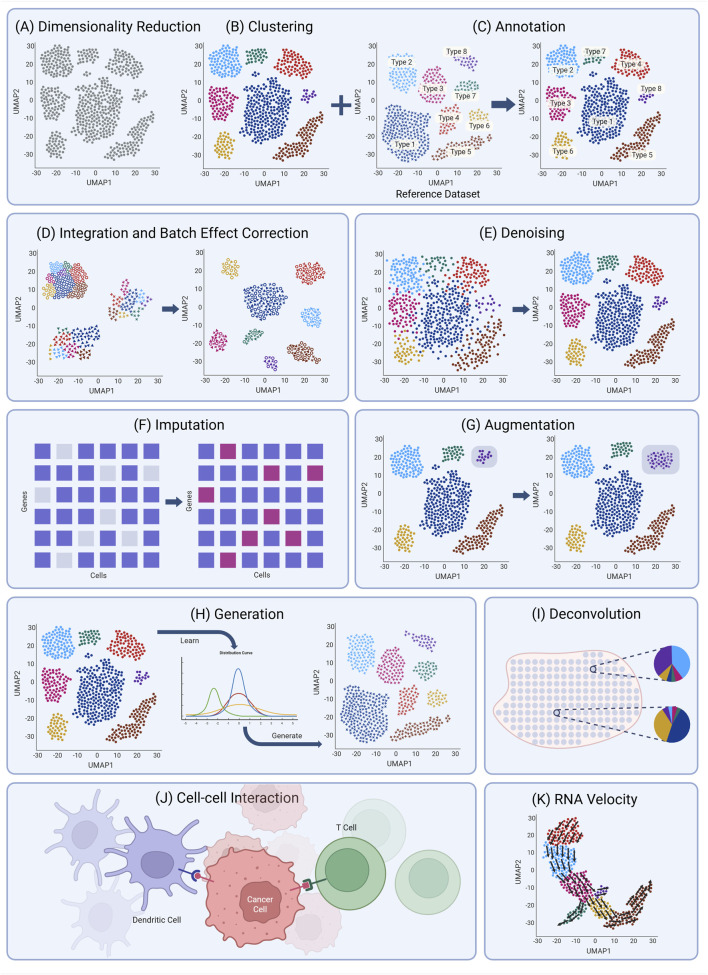
Deep learning application in single cell transcriptomics and spatial transcriptomics. **(A)** Dimensionality reduction. High-dimensional data is projected into low-dimensional space (e.g., UMAP). **(B)** Cells are clustered into distinct groups represented by different colors. **(C)** Automatic annotation of cell clusters using a reference dataset. **(D)** Integration and batch correction across different batches. **(E)** Data is denoised to recover true signal. **(F)** Data imputation to infer missing gene expression. Grey blocks (left) represent missing values, and pink blocks (right) represent imputed values. **(G)** Synthetic cells are generated to enrich rare cell type (light grey shading). **(H)** A new dataset is generated by learning distribution parameters from a reference dataset. **(I)** Each spatial transcriptomics spot is resolved into cell type fractions. **(J)** Cell-cell interactions between different cell types (e.g., dendritic cells, cancer cells, and T cells) are modeled through ligand-receptor signaling to infer intercellular communication. **(K)** Directional RNA velocity vectors are projected onto a UMAP to infer cell state transitions and lineage trajectories.

Clustering is one of the most fundamental analytical tasks in scRNA-seq and ST as it enables researchers to uncover distinct cellular populations and tissue substructures in an unsupervised, unbiased manner. Due to the, high-dimensional nature of scRNA-seq and ST data, clustering is always performed on a lower dimensional representation of the data (Figures 3A,B). Conventionally, this is PCA space (Luecken and Theis, 2019; Kiselev et al., 2019; Butler et al., 2018; Wolf et al., 2018), which is used to generate a cell-cell similarity graph, to which community detection algorithms such as Louvain (Blondel et al., 2008) or Leiden (Traag et al., 2019) clustering are applied. However, PCA assumes the lower dimensions to be linear and orthogonal and requires input data to be approximately normally distributed, thus requires pre-processing and normalization prior to use with scRNA-seq and ST data. To overcome these limitations, autoencoders (AEs/VAEs) and transformers can be used, and their learned lower dimensional embedding can be substituted for normalization and PCA in the conventional clustering pipeline. These approaches preserve the unsupervised and unbiased nature of the analysis while relaxing the assumptions and constraints required by PCA.

For scRNA-seq, a common approach is to use a VAE as implemented in scVI (Luecken and Theis, 2019; Kiselev et al., 2019; Lopez et al., 2018; Wolf et al., 2018), which incorporates a negative binomial distribution in the cost-function to model raw scRNA-seq data. Unlike most DL methods, scVI is widely used in biological analysis and is a foundation for other methods including scArches (Lotfollahi et al., 2022) and scANVI (Xu et al., 2021). In independent benchmarks, scVI embeddings are found to perform similarly to classical PCA for identification of cell types (Liang et al., 2024; Li and Quon, 2019). Other DL clustering methods for scRNA-seq include scDCC (Tian et al., 2021) and scDeepCluster (Tian et al., 2019). ScDeepCluster uses an AE architecture with a decoder that generates parameters of a zero-inflated negative binomial which is used to calculate a probabilistic loss function for scRNAseq data. scDCC extends scDeepCluster by incorporating soft pairwise constraints (e.g., must-link/cannot-link pairs derived from marker genes or protein expression) into the loss function, allowing prior biological knowledge to guide the clustering process. The method demonstrated good performance on both small (thousands of cells) and large (tens of thousands of cells) datasets, where even a few thousand constraints representing a small fraction of possible cell pairs enhanced clustering performance based on quantitative scores (e.g., Adjusted Rand Index) and more meaningful clusters than scDeepCluster, especially in difficult cases like the worm neuron dataset. However, scDCC performed similarly to state-of-the-art non-DL methods in their in-house benchmark. Whereas scDeepCluster marginally outperformed rival methods but did not compare to Louvain/Leiden clustering. Benchmarking of clustering performance is challenging due to the lack of truly orthogonal ground truth; however, these results suggest that there is no need for non-linear DL dimensionality reduction for cell type identification in scRNA-seq. In terms of applicability to biological discovery, scVI and scANVI have been used in multiple studies for dataset integration and embedding, demonstrating their utility (Salcher et al., 2022; Lindeboom et al., 2024; Yang LX. et al., 2025).

In addition to the above methods, which train a model on one specific dataset, foundation models trained on hundreds of datasets are increasingly common in scRNA-seq. Pre-trained models, such as scGPT (Cui et al., 2024) or SCimilarity (Heimberg et al., 2025) project data onto a common lower-dimensional space which could be used for clustering and novel cell type discovery. Additionally, this lower dimensional data can also be used for automatic annotation, which we will discuss further in the next section, as this space can be biased towards the most frequent cell types and miss rare cell types (Cui et al., 2024). scAtlasVAE took a foundation model approach to specifically examining T-cell heterogeneity and was able to characterize novel T-cell phenotypes when used in an unsupervised manner, identifying 18 unique and reproducible T-cell states (Xue et al., 2025).

DL approaches are also common for ST clustering due to the ease of incorporating image and/or spatial information into such models compared to the standard clustering pipeline. GCNs can incorporate spatial information by linking adjacent cells/spots into a spatial-proximity graph, leading to their use in methods such as SpaGCN (Hu et al., 2021), STAGATE (Dong and Zhang, 2022), GraphST (Long et al., 2023), SiGra (Tang et al., 2023), and DeepST (Xu et al., 2022). Similar to scRNA-seq, benchmarking studies find that DL approaches perform similarly to non-DL methods that also incorporate spatial information (Yuan et al., 2024; Hu et al., 2024a), but outperform methods that do not incorporate spatial information.

Image information is typically incorporated into ST clustering using a separate image-focused AE/VAE or GNN, which learns salient image features from individual image patches associated with the gene expression spots. These are then integrated with gene-expression features to obtain a combined embedding for each tissue spot. Although deep learning is commonly used to extract complex, high-level image features in ST clustering, some methods use non-DL approaches to integrate spatial context through hand-crafted image features. For instance, Squidpy (Palla et al., 2022) computes interpretable morphological features—such as summary statistics (mean, standard deviation), histogram-based quantiles, or textural properties (contrast, homogeneity) derived from co-occurrence matrices—for each spatial spot directly from the histology image. Similarly, SpaGCN (Hu et al., 2021) integrates image information by mapping each spatial spot to its corresponding location in the H&E image, calculating a smoothed mean RGB color value from a local pixel neighborhood, and then combining these values into a single weighted feature that reflects tissue patterns. Whereas those which use AE/VAE extracted images, gain a significant benefit from the image features, but most of the performance is driven by the gene-expression information (Tang et al., 2023; Li B. et al., 2024).

All of these methods have been demonstrated to reproduce known anatomy, but none have demonstrated a capability to identify novel, biologically meaningful structures, due to limitations in validation and ground truth availability. Thus, these approaches should be considered validated as a supplement to aid anatomical annotation by an expert. However, their capacity for novel discovery remains unknown.

Overall, AE and VAE methods for scRNAseq perform comparably to PCA and may be good alternatives when working with very large datasets. In particular, scVI has proven strong performance in many studies. For ST, DL approaches are a necessity when integrating image information into lower dimensional embeddings. GraphST is currently the best performing DL method for ST spatial domain identification.

### 3.2 Automatic annotation

Increasingly, scRNA-seq clustering is being supplemented with direct algorithmic annotation of cells with their cell type identity (Luecken and Theis, 2019) (Figure 3C). Comparing novel cells to existing annotated scRNA-seq dataset enables the inference of cell type identity through simple guilt-by-association approaches, and many early methods simply used standard similarity metrics or standard machine-learning algorithms such as support vector machines or random forests while achieving reasonably accurate results (Kiselev et al., 2018; Abdelaal et al., 2019). However, these methods tended to perform poorly on fine-scale classification of subtypes or cell-states.

DL models are highly amenable to supervised classification tasks such as cell type annotation, and, once trained, are highly efficient and scalable to millions of novel data points (Cheng et al., 2023a). Thus, dozens of novel DL models have been developed for this task using a variety of architectures, including GPT-4 and scBERT - large language models which use marker genes to annotate cells using the scientific literature (Yang et al., 2022; Hou and Ji, 2024); scGAA and TOSICA - attention-based transformer models which compare novel cells to narrow reference datasets (Chen J. et al., 2023); and pre-trained foundation models, such as scGPT (Cui et al., 2024) or CellFM (Zeng et al., 2025).

Most of these methods achieve annotation accuracies of ∼80–90%; however, in many cases, benchmarking is performed by splitting individual datasets into training and test sets, which is biased in favor of good model performance. This is because there are no systematic batch effects between the training and test data, as would be present in a real use case when these models are applied to completely novel scRNA-seq dataset (Yang et al., 2022; Cui et al., 2024; Zeng et al., 2025; Cheng et al., 2023a). Only scGPT was tested on a left-out data partition, achieving good results (accuracy >85%) for 70% of cell–types; however, performance rapidly declined as the difference between query and reference datasets increased, with fewer than 50% of cell types achieving good performance when the query dataset originated from an unseen disease state (Cui et al., 2024). Many of these methods are so recent that no independent benchmarking is available. However, in previous independent benchmarks, DL models outperformed many non-DL annotation algorithms but did not outperform a support vector machine trained on the same reference data (Kiselev et al., 2018; Chen J. et al., 2023). In these independent benchmarks, performance was found to rapidly degrade for DL models when reference data does not exactly match the query data, in agreement with the results shown for scGPT. However, DL models do show promise in their ability to accurately distinguish similar cell subtypes when provided sufficient training data (Zeng et al., 2025).

In discovery research, automatic annotation is typically used simply as a first pass, which is then manually checked and refined. Thus, even imperfect results from automatic annotation can still be useful to guide and accelerate annotation efforts (Clarke et al., 2021). Algorithms that assign a confidence score to annotations are most useful, since novel cell types may be discovered where automatic annotation has low confidence (Chen J. et al., 2023; Ergen et al., 2024). DL models naturally provide quantitative scores for annotation confidence, enhancing their utility in this use-case. In addition, as scRNA-seq resources continue to grow, approaches such as foundation models may be more easily expanded or fine-tuned to incorporate new training data compared to approaches based on traditional statistics. Thus, researchers should either use the method with training data most similar to their own, or if that is unknown we recommend scGPT for human data due to its extensive benchmarking so users can accurately assess how confident they should be in the results.

### 3.3 Integration and batch effect correction

Transcriptomic experiments often include multiple biological replicates which may be collected across multiple experimental batches, individuals, tissues, or different platforms, leading to various non-biological variations known as batch effects (Figure 3D). These technical artifacts cause identical cell types from different batches to appear distinct (Luecken et al., 2022; Chazarra-Gil et al., 2021; Tran et al., 2020). Early batch effect correction approaches, such as Combat (Johnson et al., 2007), used statistical regression to remove batch covariates. However, these methods tend to remove important biological variation unless it is specified as *a priori* within the model. To circumvent this, the next-generation of methods used techniques such as canonical correlation analysis or mutual nearest neighbors to identify shared biological variation across batches to preserve, while removing factors of variation ascribed to batch effects (Butler et al., 2018; Haghverdi et al., 2018; Hie et al., 2024). The current state-of-the-art non-DL integration method is Harmony (Korsunsky et al., 2019), which uses an iterative clustering then correction approach and is consistently among the top-performing methods in recent benchmarks (Tran et al., 2020; Antonsson and Melsted, 2024).

DL approaches to data integration modify the AE/VAE approach, as described above, to learn a ‘joint’ embedding space that captures biological groups while mixing different technical batches. A common approach to this modification is the use of adversarial learning, which penalizes the model for embeddings that leave batches separate (Hrovatin et al., 2024). Methods using this approach, such as scVI (Lopez et al., 2018), scANVI (Xu et al., 2021), and SAUCIE (Amodio et al., 2019), are not constrained by the linearity assumptions required by many non-DL methods, thus potentially enabling more efficient batch effect removal. An alternative approach uses conditional AE/VAEs which include the batch label in the joint embedding; data is then integrated by treating the batch effect as a linear transformation in the lower-dimensional space and projecting all batches onto a single reference sample or reference dataset. Prominent methods using this approach include scGen (Lotfollah et al., 2019) and scArches (Lotfollahi et al., 2022). Foundation models, such as scGPT, can also be fine-tuned to create project-specific joint embeddings. The extensive pre-training of such models includes ignoring batch effects and emphasizing conserved biology.

Despite theoretical advantages of DL methods for batch integration, they have often struggled in benchmarking studies, rarely matching the performance of Harmony (Luecken et al., 2022; Korsunsky et al., 2019; Lee et al., 2023). One potential cause of their poor performance is a tendency to over-correct and remove biological information, particularly when batches have substantially different cell type proportions (Luecken et al., 2022; Hrovatin et al., 2024). This can be mitigated by explicitly modeling cell types to ensure their preservation, as can be done for scGen and scANVI; however, since the goal of integration is usually to merge samples prior to clustering and cell type annotation, such an approach is generally limited to meta-analyses and atlasing projects.

While scRNA-seq integration can be achieved even with linear models, DL methods have been more successful when integrating multi-omics data, i.e., joint scRNA-seq and single-cell ATAC-seq (Lee et al., 2023). DL models excel at projecting different data types, such as multiome data, into similar embedding spaces, facilitating their integration (see section 3.9). This capability is further enhanced when combined with graph-based representations, which model cells as nodes and similarities or spatial relationships as edges. Graph structures enable the propagation of information across neighboring cells, effectively capturing local dependencies, preserving topology, and improving the alignment of biological states across datasets. This is particularly valuable for integrating spatial transcriptomics data or enforcing structural continuity multiple slides of the same tissue (Khan et al., 2025; Zhang C. et al., 2024). Similar to single-slide clustering performance, the top two methods for ST integration are a Bayesian statistical approach, (Li and Zhou, 2022), and a DL approach, (Long et al., 2023; Hu et al., 2024a).

While some DL methods are competitive with state-of-the-art non-DL approaches for dataset integration, there is no clear advantage to using DL for these tasks. Scalability is often cited as the main advantage of DL integration, there are several highly scalable non-DL approaches as well, including Harmony. Two non-DL approaches are consistently among top-performers in independent benchmarks: Harmony and scMerge (Luecken et al., 2022; Tran et al., 2020; Antonsson and Melsted, 2024; Lin et al., 2019). When integrating experimental replicates containing identical cell type frequencies Harmony is recommended, however, if samples contain some non-overlapping cell type scMerge is preferable (Tran et al., 2020). For atlasing and meta-analyses it can be more optimal to utilize scANVI if cell type labels are available for the respective datasets (Luecken et al., 2022). For ST data, these scRNAseq methods can be used when data is aggregated at the cell or spot level; however spatial information is lost and this often results in poor spatial contiguity of integrated clusters. For spatially contiguous ST data the Bayesian-statistics based BASS algorithm has been shown to be the best option (Hu et al., 2024). However, altering observed data can only result in a loss of information, thus integration should only be used when inspection of the data indicates substantial batch effects are present.

### 3.4 Denoising and imputation

Denoising and imputation are two closely related but conceptually distinct tasks in single-cell transcriptomics. Denoising refers to the reduction of technical noise such as amplification bias, batch effects, or stochastic dropout while preserving the true biological signal (Figure 3E). The goal is not to “fill in” missing values, but to refine observed expression levels to better reflect underlying biology. In contrast, imputation explicitly aims to predict unobserved or missing values, such as zero counts, that are likely due to technical dropout rather than true biological absence (Figure 3F). While both processes can result in modified gene expression matrices, their objectives differ: denoising aims to improve signal-to-noise ratios, while imputation attempts to recover missing information. Despite this distinction, the terms are often used inconsistently in the scRNA-seq and ST literature. Many methods described as “imputation tools” (e.g., MAGIC (van Dijk et al., 2018), scImpute (Li and Li, 2018)) perform what is effectively denoising, as they smooth expression values without necessarily distinguishing between true zeros and dropouts.

Denoising data was one of the first applications of DL models (Vincent et al., 2008). AE models have been used to denoise many types of data in various contexts; in the biomedical field, (Gondara, 2016), (Su et al., 2015), and many–omics dataset (Eraslan et al., 2019; Lal et al., 2021; Webel et al., 2024). Due to the low input material in single-cell assays, there are many missing values, and sampling- or RNA-capture-related noise is high relative to the true biological signals. Hence, many DL algorithms have been developed to denoise scRNA-seq and ST data.

One of the first and most used approaches is deep-count autoencoder (DCA) (Eraslan et al., 2019). DCA modified the traditional AE architecture to output parameters of a statistical distribution for each input gene, rather than a single predicted value. Multiple distributions are available, including negative binomial and zero-inflated negative binomial for RNA-seq data. This alteration allows DCA to account for uncertainty in the input data and biological stochasticity. Another popular method, scVI, takes a similar approach (Lopez et al., 2018). Many other model designs have been explored, including CNNs (Zhang W. et al., 2024), gene partitioning and sub-networks (Arisdakessian et al., 2019), GCNs (Huang et al., 2023), and contrastive learning (Xu et al., 2020; Shi et al., 2023). Application of these methods to biological datasets can improve the interpretability of the data; for instance, DCA increased *CD3E* expression from 80% to 99.9% in T cells and recovered *ITGAX* expression consistent with NK biology.

Only DCA, scVI, and DeepImpute have been independently benchmarked alongside non-DL denoising and imputation methods (Cheng et al., 2023b; Andrews and Hemberg, 2019; Hou et al., 2020; Huang et al., 2025). These benchmarks find conflicting results, reflecting differences in testing datasets and specific tasks used to evaluate performance. When evaluated on their ability to recover corrupted expression values or improve accuracy of automatic cell type annotation, DL denoising methods performed well, similar to other imputation and denoising methods. For unsupervised clustering and pseudotime analysis, results range from modest improvement to worse performance than the raw data, depending on the specific dataset and analysis pipeline. Whereas for gene-gene correlations, differential expression, cell type markers, and cell-cell interactions, all benchmarks find that denoising introduced a significant number of false-positive results. Hence, for scRNA-seq data, denoising remains controversial and rarely used in discovery research.

For ST data, integration with scRNA-seq is more common than direct denoising of ST data alone, which is discussed later in this manuscript. However, some methods do exist to directly denoise ST data using GNNs (Tang et al., 2023; Duan et al., 2024). Benchmarking of these methods is more limited, but SiGra is shown to increase the number of differentially expressed genes - though the extent to which these are false positives is not explored - and to improve distinctiveness of clustering. Whereas Impeller (Duan et al., 2024) is only shown to recover masked expression values.

Overall, it is not recommended to perform denoising or imputation except to enhance the sensitivity of clustering analysis, and caution must be exercised in the interpretation of results to avoid false-positives. Integration across experiments or modalities is likely a more useful task and more reliable approach for increasing statistical power by increasing the number of samples in discovery research.

### 3.5 Data generation and augmentation

Deep learning has increasingly been leveraged for data generation and augmentation in scRNA-seq and ST to address limitations posed by small sample sizes, rare cell types, and costly experimental procedures. Data augmentation in scRNA-seq and ST analysis is used differently than in machine learning and typically refers to the computational creation of additional data points, and adding them - ‘augmenting’ - to the original measured data (Figure 3G). In contrast, we will use ‘data generation’ to describe methods which create data either for the purposes of simulating data for benchmarking, or to generate data of a different modality–e.g., predict scRNA-seq from bulk RNA-seq.

In scRNA-seq, VAEs-based models like scVI and scVAE (Li and Li, 2018) can be used to generate synthetic cells that preserve the statistical properties and cellular identities of the original cell (Figure 3H). Generative models such as cscGAN (Xu P. et al., 2023) and scGFT (Vincent et al., 2008) have demonstrated the ability to generate realistic synthetic cells that preserve intrinsic gene expression profiles of the original data. Current state-of-the-art clustering and trajectory analysis algorithms, such as maximum modularity or minimum spanning trees, can be biased with respect to the number of cells, leading to poor performance when datasets include rare cell types. Selective generation and augmentation using cscGAN or scGFT can rebalance datasets, which were shown to improve clustering and trajectory inference performance to correctly identify rare cell types and accurately resolve trajectory branches. However, similar to denoising, data augmentation involves artificially amplifying the power of statistical tests, thus. are likely to result in inflated type-1 errors if used for differential expression, though this has not yet been tested.

In spatial transcriptomics, data generation is typically used for denoising purposes (Hu et al., 2021; Tang et al., 2023; Pratama et al., 2025). For instance, SiGra, discussed previously, replaces observed data with generated data to perform its denoising. Similarly, the STAGE model focuses more on accurate data generation but uses that generated data to recover and denoise down-sampled data as well as to impute between sequential ST slices (Li et al., 2024b). Both methods integrate spatial embeddings with gene expression features using autoencoders and other representation learning approaches to learn a feature space, from which new samples can be drawn and decoded into new expression data. SiGra uses both gene expression and features from matching histology, whereas STAGE uses gene expression only. Compared to single-cell RNA-seq, there are currently relatively few methods dedicated specifically to data generation and augmentation in ST. While emerging techniques focus on integrating image features, spatial coordinates, and gene expression for augmentation, these models only generate gene expression data, not matching image data, thus lacking the ability to fully generate ST data.

Similar to imputation, there is substantial risk of increasing Type-I errors when augmenting datasets with synthetically generated data. Thus, such approaches must be used with care. For data augmentation, the main utility is in facilitating detection of rare cell types or smoothing out cell density along developmental trajectories to better align data with the limitations and assumptions of the analytical tools for clustering and trajectory analysis. The only other use for data generation is for benchmarking algorithms, however, most DL generative algorithms lack the fine-scale control required to design specific ground-truth cases for that type of testing thus this area is still dominated by small-scale statistical simulation methods often custom designed for a specific benchmarking task.

### 3.6 Deconvolution

In transcriptomics, deconvolution is the decomposition of bulk expression data into cell type proportions or cell type specific expression (Im and Kim, 2023) (Figure 3I). Deconvolution is typically applied to bulk RNA-seq or low-resolution ST where each spot typically contains multiple cells. Methods for bulk RNA-seq deconvolution can be broadly grouped into statistical approaches: (Chu et al., 2022; Peng et al., 2019; Wang et al., 2019):enrichment-based methods (Aran et al., 2017; Yoshihara et al., 2013) and machine learning models (Newman et al., 2015; Newman et al., 2019). With the emergence of deep learning, at least 13 DL-based deconvolution tools have been developed for bulk RNA seq using a scRNA-seq reference (Lomas Redondo et al., 2025). These methods are typically based on multilayer perceptrons (MLPs), autoencoders, or transformers, and are trained to reconstruct cell type proportions from mixed bulk expression profiles. Scaden (Menden et al., 2020) was one of the first deep learning tools in this area. It uses an ensemble strategy that combines three deep neural networks with different numbers of layers, activation functions, and dropout settings to improve generalization. DAISM-DNN^XMBD^ (also called Aginome-XMU) instead trains a separate deep neural model for each cell type to predict proportions (Lin et al., 2022).

Bulk deconvolution methods are typically benchmarked by comparing their predictions against cell type proportions derived from *in vitro* experiments or from *in silico* bulk samples generated using single-cell RNA-seq data. Both Scaden and DAISM-DNN^XMBD^ have been independently benchmarked among the top-performing methods, with Scaden suffering high false-positive rates (Tran et al., 2023) and DAISM performing well in both coarse-grain and fine-grain deconvolution (White et al., 2024). This demonstrates that deep learning provides a strong alternative to traditional approaches. Newer methods may outperform DAISM, but this cannot be established until a systematic benchmark study has been performed that includes the other DL-based deconvolution tools.

Overall, bulk RNA-seq deconvolution enables researchers to reduce experimental costs while still gaining insight into the tumor or tissue microenvironment. However, the performance of DL deconvolution methods requires high quality training dataset and is prone to poor generalization (Wolfram-Schauerte et al., 2025). Most researchers still rely on traditional deconvolution approaches, and only a few studies have utilized DL-based tools for deconvolution (Chen et al., 2025; Codino et al., 2025; D’Sa et al., 2025).

Bulk RNA-seq deconvolution tools can be used for ST data, but additional improvements in performance may be achieved by incorporating the spatial information. Many ST deconvolution methods use non-DL approaches such as numerical optimization (Dong and Yuan, 2021), or probabilistic models (Kleshchevnikov et al., 2022). Several DL-based deconvolution methods not only estimate the cell type fractions but can also estimate the number of cells per spot, generate gene expression for each deconvolved cell, or estimate individual cell locations (Gaspard-Boulinc et al., 2025).

Reference-based DL deconvolution methods use three general strategies: supervised-learning, similarity-based integration, and foundation models. Supervised-learning creates synthetic ST spots by combining scRNA-seq data and use this as ground truth to train a neural network to predict cell type fractions from the aggregated expression profile (Lund et al., 2022; Bae et al., 2022; Zhan et al., 2025; Xu H. et al., 2023; Mañanes et al., 2024). Similarity-based integration methods embed scRNA-seq and ST data into a shared space through graph construction (Long et al., 2023; Ding et al., 2024; Song and Su, 2021; Li and Luo, 2024; Yin et al., 2024; Zhang et al., 2023), autoencoders (Liao et al., 2022; Hao et al., 2024c; Coleman et al., 2023; Li H. et al., 2022), or optimization (Biancalani et al., 2021) to match ST spots to scRNA-seq cell types based on similarity or distance measures. In some methods, pseudo-spots are generated to aid embedding (Ding et al., 2024; Song and Su, 2021; Li and Luo, 2024; Yin et al., 2024; Zhang et al., 2023; Li H. et al., 2022). UniCell Deconvolve (UCD) is the only foundation model trained for deconvolution (Charytonowicz et al., 2023). It is a feedforward neural network trained on over 840 cell types from 899 single cell datasets. UCD uses transfer learning to adapt the foundation model to specific context where users have an option to input a contextualized reference profile to fine-tune a regression model using UCD base embedding. UCD outperformed other methods on synthetic mixtures from its own training data, but had only average performance on out-of-sample tests unless it was fine-tuned on the relevant datasets (Charytonowicz et al., 2023). An alternative approach is taken by scResolve, which imputes pixel-level gene expression which is combined with cell-segmentation of the respective histology image to infer single-cell resolution expression (Chen H. et al., 2023). This enables reference-free deconvolution and potentially novel cell type discovery.

Due to the wide variety of spatial deconvolution tools, no systematic benchmark study has yet been conducted across all methods, and most DL-based approaches have not been benchmarked. Benchmarking is especially challenging in ST deconvolution since ground truth is not available; instead, simulated ST datasets generated from scRNA-seq are typically used. Tangram (Biancalani et al., 2021) and DSTG (Song and Su, 2021) have been benchmarked in multiple independent studies alongside non-DL methods (Li et al., 2023; Chen J. et al., 2022; Yan and Sun, 2023; Li B. et al., 2022). While Tangram was shown to be superior in predicting the spatial distribution of transcripts in one study, both Tangram and DTSG generally ranked within the top third of approaches benchmarked. However, the top three performing methods overall were non-DL approaches. DL methods have the advantage of integrating multimodal data, such as histology images, which may provide additional information such as cell morphology to aid deconvolution.

For discovery focused researchers cell2Location (Kleshchevnikov et al., 2022) and SpatialDWLS (Dong and Yuan, 2021) remain top choices for deconvolution when reliable reference single-cell datasets are available. Tangram is an acceptable alternative, and scResolve is the only method capable of deconvolution when no reference single-cell data is available.

### 3.7 Cell-cell interactions

A key goal of single-cell RNAseq was to identify interactions between different cell types which would normally be obscured in bulk tissue samples. Many heuristic methods have been developed for this task, including CellChat (Jin et al., 2021), CellPhonedb (Efremova et al., 2020), SingleCellSignalR (Cabello-Aguilar et al., 2020), and NicheNet (Browaeys et al., 2020), which use databases of ligand-receptor (LR) pairs and calculate a co-expression score of each pair between pairs of cell types. Some of these have been expanded to account for spatial location, for use with spatial transcriptomics (Efremova et al., 2020; Dimitrov et al., 2024). Currently, there are only a few DL approaches to inferring these interactions in single cell data and none for spatial transcriptomics.

DeepCCI (Yang et al., 2023a) integrates ResNet and a GCN model to infer cell-cell interactions with a common decoding layer. This decoding layer is trained using consensus interactions obtained from the heuristic methods. As a result, in their in-house benchmarking DeepCCI identifies the same interaction as multiple heuristic methods though may have fewer false-positive results than any of the heuristic methods used alone. It is unclear whether DeepCCI gains anything from the DL components, as opposed to their in-house consensus of the heuristic models used to train it.

An advantage of DL approaches is the ability to integrate multiple data sources; this is utilized by GraphComm (So et al., 2025) to integrate pathway annotations in addition to direct LR interactions into a prior interaction probability between each LR pair. Coexpression of LR pairs is calculated and is integrated with the prior using a graph attention network. The embedding contains both cell types and LR genes and is used to generate LR pairwise scores and cell type x cell type scores by multiplying the respective embeddings. Alternatively, ScTenifoldXct (Yang Y. et al., 2023) and scSDNE (Jia et al., 2025) first infer gene-gene dependencies either using a DL model (scSDNE) or a regression model (ScTenifoldXct), which is combined with a LR coexpression score which is then used to generate a gene embedding space using a graph-autoencoder architecture. Cell-cell interactions are inferred from proximity of LR pairs in the gene embedding space. ScSDNE and ScTenifoldXct have the advantage of using semi-supervised learning, whereas GraphComm relies on database-derived LR interactions to train their embedding space. Limited in-house benchmarking is available for these, but they perform similarly to heuristic methods, with GraphComm seeming to have higher sensitivity, whereas scSDNE and ScTenifoldXct are more conservative, performing similar to a consensus of heuristic methods.

Cell-cell interaction inference remains challenging, primarily due to the lack of any true gold-standard benchmarks. In many cases, methods are benchmarked using spatial transcriptomics data, as distant cells are unlikely to interact, but this cannot provide individual LR interaction information, or with very small sets of manually curated interactions. This is particularly problematic for DL algorithms due to their reliance on training data to optimize the models. Typically, researchers use multiple LR algorithms and use some kind of consensus as evidenced by the popularity of the LIANA package (Dimitrov et al., 2024). The natural ability of DL to integrate multiple types of data may be an advantage here, as significant amounts of perturbation data are available which could potentially be used to augment cell-cell interaction inference. However, currently there is little evidence due to lack of gold-standard datasets to favour any specific method over any other.

### 3.8 Combining single-cell and spatial transcriptomics

ST and scRNA-seq are complementary techniques; scRNA-seq accurately assesses the entire transcriptome for each individual cell but it loses all spatial information, whereas in ST spatial information is preserved but either data is not at single-cell resolution and/or does not capture the entire transcriptome. As a result, many methods have been developed to combine scRNA-seq and ST using different approaches. SIMO uses optimal-transport to align single cells to ST based on only RNAseq or both RNA and ATACseq modalities (Yang P. et al., 2025), Alternatively CellTrek (Wei et al., 2022) uses mutual-nearest-neighbour integration combined with random forests to predict spatial location of individual cells from proximity within the integrated embedding space. In in-house benchmarking CellTrek performed well on simulated ST data but was not compared to DL alternatives.

One of the first and most established models is Tangram, which learns a mapping between scRNA-seq and ST that optimizes the spatially correlation between mapped and observed gene expression (Biancalani et al., 2021). The authors demonstrate its effectiveness in recapitulating known expression patterns across cortical layers. In independent benchmarks, Tangram out-performs other methods for recovering downsampled gene expression values but shows modest performance at predicting cell type composition of ST data (Li B. et al., 2022). However, notably neither the original publication nor independent benchmarks assessed potential for generation of false-positive results. Generative DL models can predict scRNA-seq profiles from ST data based on a reference scRNA-seq dataset. For example, SpatialScope uses a probabilistic DL model to predict cell type composition of individual ST spots and to decompose gene expression by cell type, and then uses a generative DL model to create scRNA-seq for individual cells based on the decomposed profiles (Wan et al., 2023). In contrast, stImpute predicts gene expression for unmeasured genes in imaging-based ST using a joint AE embedding and GNN, based on known gene-gene relationships (Zeng et al., 2024).

Prediction of additional data modalities or higher resolution data from cheaper, lower resolution experimental protocols is a popular use-case for DL method development. ScSemiProfiler predicts scRNA-seq from bulk RNA, which has the advantage of being able to predict cell type specific differences in expression which is not possible with non-generative deconvolution methods (Wang et al., 2024). Using matched bulk and scRNA-seq data from COVID-19 patients, the authors were able to show their method could capture individual difference beyond what was present in the training data. However, they did not evaluate whether scSemiProfiler’s cells would lead to the same biological conclusions on the effect of COVID-19 as the original scRNA-seq. Thus, it remains unclear if this approach is viable for discovery research.

Lastly, over a dozen algorithms have been published that predict ST expression data from histology images. Histology images are plentiful and easily collected, whereas ST is relatively rare and expensive; therefore, accurate prediction of the latter from the former would be very valuable. However, performance of all current methods is relatively poor with correlations between predicted gene expression and true measured gene expression below 0.2 for most genes (Wang et al., 2025). While performance is best for genes with strong spatial patterning, correlations remain below 0.5 in nearly all cases, still far below an accuracy that would be useful for discovery research. Such methods may improve as ST experimental platforms improve, though it is also possible that much of gene expression does not manifest as any visible difference in histology images, thus placing a hard limit on the maximum accuracy of these methods. The most likely limitation of current models, however, is the availability of ST training data with high quality matching histology images as most publicly available data only release a compressed low-resolution image.

Overall, discovery researchers are recommended to choose methods which project single-cells onto ST data rather than any generative approaches, such as SIMO or CellTrek, and to use multiple different methods to ensure conclusions are robust to the approach chosen. While generative DL approaches are promising for converting between transcriptomic technologies, there is insufficient benchmarking in real-world use cases to know whether these methods lead to false or misleading conclusions.

### 3.9 Integrating multiomic data

ST data can be considered multiomic in that images and spatial coordinates can be treated as another layer of data to be integrated. However, more often multiomic data refers specifically to single-cell data where both mRNA is captured and sequenced and DNA is capture either for direct DNA sequencing or most often for ATAC assays, which measure open chromatin across the genome (Mimitou et al., 2021; Cao et al., 2018; Reyes et al., 2019). While first developed for single cells, equivalent assays have been developed for spatially-resolved assays (Jiang et al., 2023; Guo et al., 2025; Deng et al., 2022). However, currently only simultaneous single-cell RNA-seq and ATAC-seq has been developed into a simple off-the-shelf platform, thus is by far the most used multiome technique.

Popular methods for single-cell multiome (scMultiome) data integration and analysis include ArchR (Granja et al., 2021), Signac (Stuart et al., 2021), and MOFA (Argelaguet et al., 2020) which perform data normalization, dimensionality reduction, and clustering. Signac and ArchR in addition identify correlated open-chromatin peaks and nearby gene-expression which can be used to infer gene-regulatory networks. These approaches are all statistical approaches, with ArchR and Signac both using latent semantic indexing for data embedding, and MOFA using a Bayesian probabilistic model for joint factor analysis.

DL approaches have several advantages for multiomic data integration. They can innately align different input data such that ATAC peaks do not have to be assigned to genes prior to integration. They can be regularized to learn comparable representations for different modalities from the data rather than using heuristic normalization strategies. Finally, the architecture can be data-type invariant allowing the same structure to be used for many different data modalities. The general structure of DL multiome methods starts with modality-specific AEs or VAEs then combines the modality-specific embeddings into a single representation (Ashuach et al., 2023; Gong et al., 2021; Li G. et al., 2022; Cao and Gao, 2022).

MultiVI (Ashuach et al., 2023) uses this approach to expand the scVI architecture to multiome data by penalizing the model for divergent representations for the same cell in different modalities then using the average representation for each cell. This enables efficient integration of paired and unpaired datasets since unpaired data simply uses the single representation value. Cobolt (Gong et al., 2021) has a very similar architecture but uses a Dirichlet prior and reconstructs the original matrices rather than using the decoder to estimate the original distribution. scMVP (Li G. et al., 2022) has the same overall architecture but uses self-attention and mask-attention encoders for each modality and simply concatenates the latent spaces for the joint embedding. Whereas GLUE (Cao and Gao, 2022) uses heuristic methods to infer ATAC-peak to RNA-gene associations which are used as knowledge graph as an additional decoder output from the concatenated multiomic latent space of their AE.

In multiple independent benchmarks (Xiao et al., 2024; Liu et al., 2025; Hu et al., 2024b; Fu et al., 2025), Seurat’s weighted nearest network (WNN) consistently output performs other integration methods in perfectly matched RNA + ATAC data, whereas MultiVI is consistently optimal for partially overlapping datasets. In contrast, GLUE is the best performer when ATAC and RNA datasets are from separate samples. Notably, these results were simply for the level of integration of the lower dimensional embedding, i.e., the mixing of ATAC and RNA modalities while preserving or enhancing cell type identities. One benchmark (Hu et al., 2024b) evaluated modality prediction, and while MultiVI was a top performer, all methods had relatively poor performance (correlation <0.4) generally due to overestimation for genes upregulated in a particular group of cells, this is in line with other benchmarking of imputation methods where data smoothing typically inflates signals resulting in false-positives (Andrews and Hemberg, 2019).

Overall MultiVI and GLUE are both established methods with strong performance in benchmarks and would be good choices especially for projects with not completely overlapping scMultiome data. Heuristic methods, particularly Seurat’s WNN method, are good choices for perfectly matched datasets but are inadequate for non-overlapping datasets. Imputation is still unreliable and should not be used for statistical analyses, though may be useful for identifying trends for independent validation. While DL algorithms have been developed for integration and imputation of scMultiome, inference of gene-regulatory networks which is often the main goal of Multiome studies has not yet been addressed with DL methods and may be an opportunity for future method development.

In an independent benchmark on curated datasets, scJoint, MultiVI and GLUE were top performing methods for integrated cell type identification in scMultiome data (Xiao et al., 2024). However others find high variability in performance dataset to dataset and that MultiVI was particularly sensitive and either were among top performers or worse performers depending on the dataset in question (Lee et al., 2023).

For spatial multiome, many of the above single-cell methods would be applicable; however, when spatial data includes contiguous homogeneous regions, it is often beneficial to incorporate spatial information as we noted above. Currently, the only method that integrates spatial location for spatial multiome data is SpatialGlue (Long et al., 2024). This method encodes spatial information as a graph linking spatially proximal cells or spots and uses an AE structure to learn a joint embedding space. To integrate RNA and ATAC data, separate GCN encoders combine the spatial graphs with modality-specific similarity graphs. These encodings are combined with an attention head to generate a single embedding space across both spatial modalities. In-house benchmarking on datasets with known anatomical regions showed good performance compared to non-spatial statistical or DL models. In agreement with ST vs. scRNA-seq data analysis, significant improvements in identifying spatial regions can be achieved by incorporating physical proximity, and DL models are more easily adapted to include this information than statistical methods.

### 3.10 RNA velocity

While scRNA-seq provides a snapshot of transcriptional states, RNA velocity methods have become increasingly valuable tools for investigating cell trajectories (Shima and mura, 2025; Bergen et al., 2021; Ge et al., 2025). Although new, several computational approaches now exist that leverage the relative abundances of spliced and unspliced mRNA to quantify transcriptional dynamics. Early ordinary differential equation (ODE)-based approaches like velocyto assumed specific cells were near steady-state, whereas scVelo relaxed this assumption through maximum-likelihood inference (La Manno et al., 2018; Bergen et al., 2020). More recent approaches incorporate additional molecular information, such as chromatin accessibility and protein expression, thereby refining trajectory inference and interpretability (Luo et al., 2025).

Recently, DL-based RNA velocity models have emerged to better capture nonlinear transcriptional dynamics and complex cellular transitions (Ge et al., 2025; Luo et al., 2025; Gayoso et al., 2024). VeloAE employs an autoencoder architecture to learn denoised, low-dimensional representations of RNA velocity (Qiao and Huang, 2021). VeloVAE and VeloVI employ VAE frameworks to infer RNA velocity and jointly quantify uncertainty (Gayoso et al., 2024; Gu et al., 2022). VeloVAE models a shared developmental timeline across all cells by learning latent time and cell-state representations, enabling explicit modelling of cell-fate branching and differentiation pathways. Conversely, VeloVI fits gene-specific dynamical models by leveraging information across cells, offering robust and reliable uncertainty estimates for RNA velocity at both gene and cell levels. DeepVelo integrates a graph convolutional network with a VAE to model gene- and cell-specific transcriptional kinetics, improving accuracy across heterogeneous cell populations (Chen Z. et al., 2022; Cui et al., 2023). LatentVelo and cellDancer both utilize neural architectures; LatentVelo embeds cell states and velocities into a latent space, while cellDancer employs gene-specific networks that aggregate local neighborhood information to infer cell- and gene-level kinetics (Li et al., 2024c; Farrell et al., 2023).

Regarding benchmarking, the accuracy and stability of these methods remain variable across datasets (Bergen et al., 2021; Luo et al., 2025; Gayoso et al., 2024; Gorin et al., 2022). Though deep learning approaches often perform better on complex datasets, no single method excels in both accuracy and stability (Shima and mura, 2025; Gayoso et al., 2024). Accuracy measures how closely predicted velocities align with known or expected biological trajectories. However, benchmarking remains limited due to limited ground truths, thus relying on indirect metrics based on velocity cosine similarity and agreement with known lineages (Bergen et al., 2021; Luo et al., 2025; Gayoso et al., 2024). Although most methods displayed locally consistent velocities between neighboring cells, most fail to reliably infer true cell-state transitions, particularly in complex or branching trajectories (Luo et al., 2025; Qiao and Huang, 2021; Gorin et al., 2022; Ancheta et al., 2024). In addition, discrepancies between methods remain common, primarily due to differences in model assumptions and datasets used (Bergen et al., 2021; Luo et al., 2025; Gayoso et al., 2024; Ancheta et al., 2024). Downsampling had the greatest impact on ground-truth recovery, while inter-method consistency remained stable. (Shima and mura, 2025; Luo et al., 2025; Ancheta et al., 2024). Notably, DeepVelo, scVelo, VeloVI, and velocyto often showed higher agreement among themselves, but none stood out in either accuracy or consistency across datasets.

In discovery contexts, current RNA velocity approaches should be interpreted cautiously when resolving complex cell-state transitions (Bergen et al., 2021; Gorin et al., 2022) Methods like VeloVI and LatentVelo offer higher accuracy and stability in specific contexts, but none are universally dependable (Luo et al., 2025; Gayoso et al., 2024). Using multiple RNA velocity methods in combination can mitigate individual biases, while integrating multi-omic or lineage-tracing datasets can help correct technical biases by providing more reliable validation (Shima and mura, 2025; Bergen et al., 2020; Mao et al., 2025). As the field of RNA velocity advances, deep learning methods will become more robust, capturing transcriptional kinetics from diverse datasets and reducing dependence on traditional ODE assumptions.

## 4 Conclusion

A plethora of algorithms and software packages have been produced using DL to solve many common problems in scRNA-seq and ST analysis. However, the performance of these models has been variable, with only the top models being competitive with state-of-the-art non-DL alternatives. There is no evidence that DL is inherently more accurate than non-DL algorithms, nor is it inherently more scalable when compared to optimized non-DL approaches. While DL can remove the linearity assumptions that constrain alternative approaches, there is little evidence that this provides a substantial benefit. The advantage of DL algorithms is their flexibility in handling a wide range of data types, which enables simple approaches for combining different data modalities, while graph-based models can be easily used to incorporate a spatial dimension. In addition, generative DL can enable novel approaches, mainly the prediction of one data modality from another, that are not easily amenable to non-DL models. However, it remains to be proven that such algorithms can reach sufficient precision for their use in discovery research.
